# Phytochemicals, Extraction Methods, Health Benefits, and Applications of Loquat (*Eriobotrya japonica* Lindl.) and Its By‐Products: A Comprehensive Review

**DOI:** 10.1002/fsn3.70483

**Published:** 2025-07-01

**Authors:** Xiaofeng Liu, Xiulei Cai, Zhiyu Chen, Yao Zhang, Hao Zhong, Rongfa Guan

**Affiliations:** ^1^ College of Food Science and Technology Zhejiang University of Technology Hangzhou Zhejiang China; ^2^ Zhejiang Provincial Key Lab for Chem and Bio Processing Technology of Farm Produces, School of Biological and Chemical Engineering Zhejiang University of Science and Technology Hangzhou Zhejiang China

**Keywords:** active substances, biological activity, extraction mode, loquat and by‐products, mechanism of action

## Abstract

Loquat ( *Eriobotrya japonica* ), a plant native to China and having extensive medicinal values, has recently drawn considerable attention. The current research on loquat mainly centers on its preservation, processing, and extraction of bioactive compounds. Nevertheless, there are still challenges and problems in the medical research and application of loquat and its byproducts. This study intends to explore the pros and cons of various methods for extracting abundant bioactive substances from loquat and its byproducts. The crucial antioxidant, anti‐inflammatory, and hypoglycemic roles of these bioactive substances and their significance in the domains of medicine and healthcare were evaluated. A thorough examination of the functions and mechanisms of action of the bioactive compounds in loquat will provide new insights and opportunities for the enhancement of the medicinal potential of loquat. It will also act as an important reference for research and application in related fields.

## Graphical abstract

Loquat and its by‐products are good sources of various bioactive compounds. Loquat extract has a series of health‐promoting effects. The development and application of loquat have broad prospects.

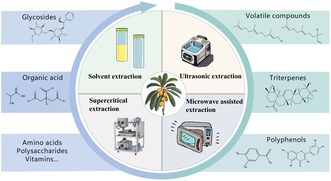

**Abbreviations**
AKT: protein kinase B
AMPK: AMP‐Activated Protein Kinase
CYP2E1: Cytochrome P450 2E1
ERK: extracellular regulated protein kinases
FOXO3: Forkhead box O3
GLUT4: Glucose Transporter 4
GSP: Glycated serum protein
IgE: Immunoglobulin E
IKK: IκB kinase
iLRS‐1: Insulin/IGF‐1‐like receptor substrate‐1
IRS‐1: Insulin Receptor Substrate‐1
LKB1: Liver kinase B1
LPS: Lipopolysaccharide
MAPKs: Mitogen‐Activated Protein Kinases
MEK: mitogen‐activated protein kinase kinase
mTOR: mammalian target of rapamycin
NDES: Natural Deep Eutectic Solvents
NF‐κB: Nuclear Factor‐κB
NLRP3: NOD‐Like Receptor Family Pyrin Domain‐Containing 3
Nrf2: Nuclear Factor Erythroid 2‐Related Factor 2
OVA: Ovalbumin
PGC: Peroxisome proliferator‐activated receptor gamma coactivator
SIRT6: Sirtuin‐6
TC: Total cholesterol
TG: Triglyceride
TRPV1: Transient Receptor Potential Vanilloid 1
TSGs: Total Sesquiterpene Glycosides
ZO‐1: Zonula Occludens‐1

## 1 Introduction

Loquat ( *Eriobotrya japonica* Lindl.) is a member of the *Eriobotrya* genus within the subfamily Maloideae of the Rosaceae family. Native to China, it is extensively cultivated in southern regions and globally, including Japan, the United States, India, Spain, Brazil, Mexico, Argentina, and Italy, among others (Hanif et al. 2019; Zhu et al. 2021; Dhiman et al. 2022). In China, loquat flowers and leaves are frequently utilized as medicinal herbs, purportedly offering benefits such as wind dispersal, cough relief, and nasal decongestion. They are traditionally employed for symptoms such as headaches, nasal obstruction, rhinorrhea, persistent cough due to fatigue, and blood‐streaked sputum. At present, loquat extracts are used for treating inflammation, diabetes, chronic bronchitis, and cancer (Liu et al. 2016). Modern purification technology has successfully isolated multiple effective components from loquat, including volatile oils such as nerolidol and farnesol, as well as tartaric acid, ursolic acid, oleanolic acid, amygdalin, ellagitannin, B vitamins, vitamin C, sorbitol, and other ingredients (Shi et al. 2018; Park et al. 2019; Sagar et al. 2020; Xue et al. 2022; Huang, Guo, et al. 2023; Huang, Liu, et al. 2023). Research indicates that these bioactive compounds possess therapeutic potential against diverse diseases, highlighting their substantial medicinal and health‐promoting attributes.

Contemporary research on loquats has focused predominantly on fruit preservation, food processing, and the extraction of beneficial components. However, loquat processing faces challenges due to the robust postharvest physiological activity of loquat fruits, which leads to rapid senescence and increased susceptibility to spoilage and decay at ambient temperatures (Wang, Wen, et al. 2023; Wang, Zheng, et al. 2023; Zhang et al. 2023). While low‐temperature storage can effectively extend the shelf life and mitigate decay, it often results in challenges such as difficulty in peeling the skin, lignification and browning of the flesh, as well as a coarse texture with reduced juiciness. These issues are the main reasons for the loss of commerciality and the reduced appeal of loquats (Zhu et al. 2018; Wang et al. 2022; Su et al. 2024). On the other hand, improper handling during processing may lead to nutrient loss. Additionally, addressing the instability of key functional components in processed products remains a significant challenge (Qiao et al. 2024). Currently, most loquat seeds generated from processing are underutilized and frequently discarded as waste in various regions. In the meantime, loquats blossom during winter, with a prolonged flowering phase lasting up to 3–4 months, where each inflorescence may yield 80–120 flowers (Zheng et al. 2015), necessitating floral thinning to maintain quality, leading to substantial floral wasting. Economically speaking, the development of the loquat deep‐processing industry has been sluggish, with only marginal improvements observed in product value addition. Delving into the active substances of the entire loquat plant aligns with the emerging “return to nature” paradigm in pharmaceutical science, which is beneficial for enhancing the market economic value of the loquat industry.

## 2 Phytochemicals in Loquats and Byproducts

**FIGURE 1:**
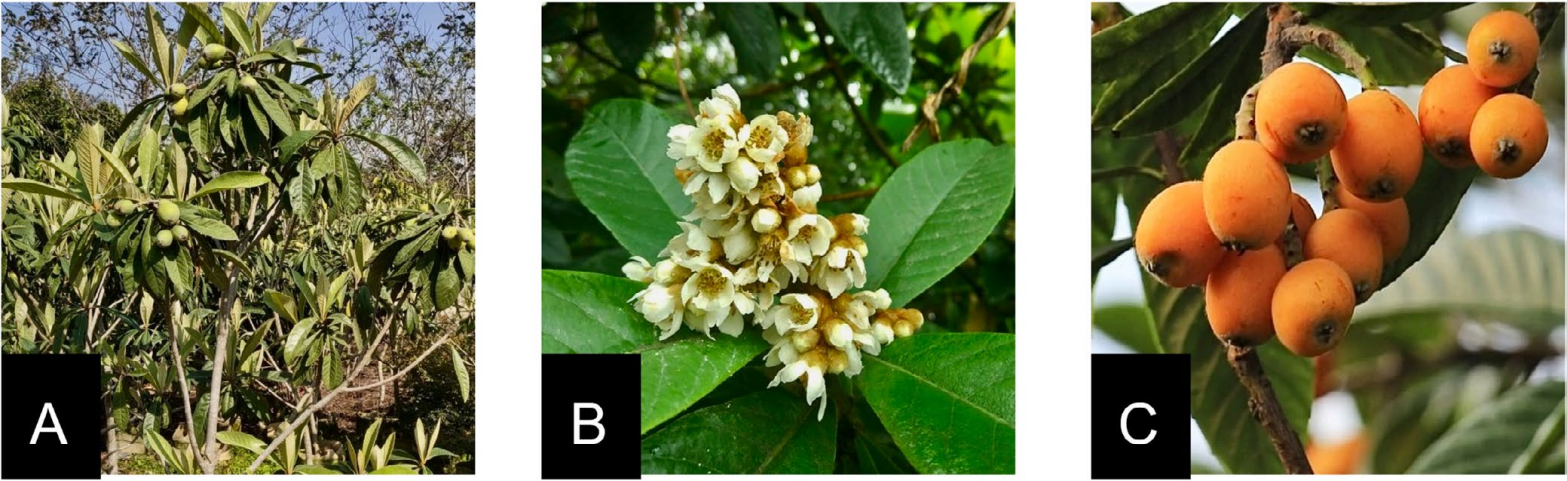
Photographs of various parts of loquat. (A) Loquat; (B) flowers and leaves; (C) ripe fruits.

Phytochemicals are low‐molecular‐weight end products of plant metabolism that play a variety of roles in plants. Although these substances are not essential nutrients, studies have shown that they play a variety of physiological roles that are beneficial to human health (Górniak et al. 2018; Denaro et al. 2020; Sangiovanni and Dell'Agli 2020). Current research on phytochemicals in loquat focuses on loquat fruits, leaves, and flowers. Among these, loquat leaves are quite popular among scientists. Loquat and its byproducts are rich in polyphenolic compounds (Figure 1) such as phenolic acids and flavonoids; volatile oils such as nerolidol and acacia alcohol; and triterpenoids such as tartaric acid and ursolic acid, glycosides, tannins, and other bioactive substances (Shi et al. 2018; Dhiman et al. 2022; Xiao et al. 2023; Raza et al. 2025). Information on the extraction and structural characterization of these compounds, as well as their biosynthetic pathways and biotransformations, can help us better understand how plants interact with their environment through these chemicals and explore their potential to promote human health (Lee et al. 2004; Martel et al. 2019; Padhi et al. 2021; Beaver et al. 2025).

### 2.1 Polyphenol Compounds

Polyphenolic compounds are among the most important secondary metabolites in plants and are widely distributed. The polyphenols contained in loquats (Table 1) can be categorized into phenolic acids, flavonoids, lignans, coumarins and hydroxycinnamic acids, stilbenes, and proanthocyanidins (Xiao et al. 2023). The study indicated that the flavonoids in loquats mainly include fourteen kinds of flavonoids, such as kaempferol, quercetin, galangal, nerol, and rutin, and fourteen kinds of phenolic compounds, including chlorogenic acid, caffeic acid, ferulic acid, gallic acid, protocatechuic acid, and sixteen other phenolic compounds (Sagar et al. 2020).

**TABLE 1:** Polyphenol compounds and contents in loquat.

Substance	Detection mode	Content	References
5‐ caffeoylquinic acid	UPLC	32.92–90.70 mg/100 g	Ding et al. (2001); Zhou et al. (2011); Zhang et al. (2015); Sagar et al. (2020)
3‐caffeoylquinic acid		9.55–20.70 mg/100 g	
4‐caffeoylquinic acid		0.52–4.33 mg/100 g	
5‐feruloylquinic acid		2.81–14.52 mg/100 g	
Hydroxybenzoic acid		2.24–8.15 mg/100 g	
4‐O‐caffeoylquinic acid	HPLC‐DAD/LC‐ESI‐M	3.13–6.75 mg/g	Xu et al. (2014); Zhang et al. (2015)
5‐O‐caffeoylquinic acid		0.46–5.10 mg/g	
3‐p‐coumaroylquinic acid		0.00–0.52 mg/g	
Neochlorogenic acid	HPLC‐DAD	310.09–1139.91 μg/g	Xu et al. (2014); Zhang et al. (2015)
Chlorogenic acid		651.92–216.65 μg/g	
4‐hydroxybenzoic acid		56.27–210.86 μg/g	
Caffeic acid		6.44–10.29 μg/g	
Ellagic acid		31.76–60.49 μg/g	
Quercetin‐3‐O‐neohesperidoside	HPLC‐DAD‐MS	/	Ferreres et al. (2009); Sagar et al. (2020)
Quercetin‐3‐O‐sambubioside		/	
Quercetin‐3‐O‐galactoside		/	
Kaempferol‐3‐O‐neohesperidoside		/	
Kaempferol‐3‐O‐sambubioside		/	
Quercetin‐3‐O‐glucoside		/	
Kempferol‐3‐O‐rhamnoside		/	
Azelechin	Chromatographic analysis	/	Ito et al. (2000)
Catechin			
Naringenin‐8‐C‐rhamnoglucoside			

The types and contents of flavonoids and phenolic acids present in different parts of loquat fruits are not the same, and notably, in loquat fruits, flavonoids are present only in the pericarp (Pareek et al. 2014). Xu et al. determined the contents of nine phenolic compounds in loquat fruits by HPLC, and the results showed that chlorogenic acid is the dominant phenolic compound in mature loquat fruits (Xu et al. 2014). The main polyphenolic compounds in leaves are phenols and flavonoids, and most of the wild varieties have high total phenolic and flavonoid contents as well as strong antioxidant activities (Hong et al. 2008). Ito et al. (2000) isolated 3 flavonoid glycosides and 15 flavonoids from loquat leaves. They also found that they are characterized by (2S)‐ and (2R)‐naringenin 8‐C‐alpha‐L‐rhamnopyranosyl‐(1 → 2)‐beta‐D‐glucopyranosides and cinchonain Id 7‐O‐beta‐D‐glucopyranoside. Tao et al. (2022) found that proanthocyanidins from loquat leaves consist of catechin, gallocatechin, gallocatechin gallate, and afzelechin with B‐type link along with a small portion of A‐type link. Subsequent studies (Nawrot‐Hadzik et al. 2017) have shown that cinchonin IIb, flavonoid glycosides, and two protocatechuic acid derivatives are the main substances with antioxidant activity. A related study (Zhou et al. 2011) showed that the content and activity of flavonoids also varied among different varieties of loquat flowers. Flowers of stage 3 had the highest flavonoid and phenolic content and showed the strongest antioxidant capacity. These results provide valuable information for a better understanding of the chemical composition of loquat. They also provide a scientific basis for further research and exploitation of the polyphenolic compounds in loquat.

### 2.2 Triterpenoid Compounds

Triterpenoids are a class of natural products formed by the polymerization of six isoprene units. They usually contain 30 carbon atoms, but derivatives with 27 or 24 carbon atoms are sometimes found (Table 2). These compounds are widespread, especially in the plant kingdom. They exist in the free state and can also be combined with sugars to form glycosides or esters. A variety of triterpenoid compounds, such as ursolic, corosolic, rosmarinic, commiphoric, oleanolic, and masuric acids, have been identified in loquat (Xue et al. 2022).

**TABLE 2:** Triterpenoid compounds and contents in loquat.

Substance	Detection mode	Content	References
2α‐Hydroxyursolic acid	Rversed phase HPLC	10.8 mg (total 399 mg)	Taniguchi et al. (2002); Xue et al. (2022); Yan et al. (2023)
Maslinic acid		11.5 mg (total 399 mg)	
Tormentic acid		45.1 mg (total 862 mg)	
2α,19α‐Dihydroxy‐3‐oxo‐urs‐12‐en‐28‐oic acid		23.6 mg (total 862 mg)	
3‐O‐cis‐p‐coumaroyltormentic acid		17.2 mg (total 717 mg)	
3‐O‐trans‐p‐coumaroyltormentic acid			
Hyptadienic acid		5.1 mg (total 862 mg)	
Alphitolic acid	LC–MS	/	Tan et al. (2017); Xue et al. (2022)
Corosolic acid		/	
Euscaphic acid		/	
Rosamultic acid		/	
Oleanolic acid		/	
Roxburic acid		/	
Asiatic acid		/	
Ursolic acid		/	
3β‐hydroxyl‐21β‐acetoxyl‐urs‐12‐en‐28‐carboxylate	Thin layer chromatography, LC–MS	15.0 mg (total 3.5 g)	Tan et al. (2017)
Sinapic acid	UPLC		Yan et al. (2023)

**FIGURE 2:**
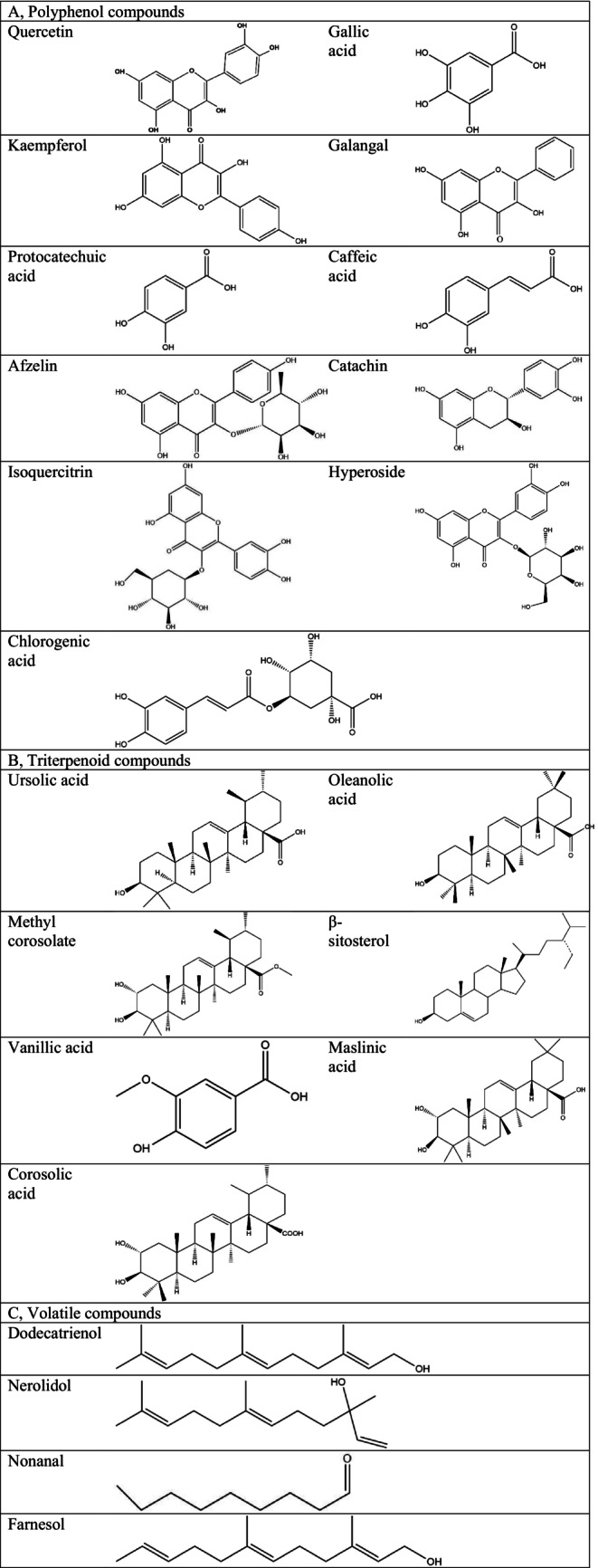
The structure of active substances in loquat and its byproducts.

Triterpenoids have been proven to have various biological activities, including lowering blood sugar, improving kidney damage, alleviating rheumatoid arthritis, and providing antiviral effects (Serra et al. 1994; Qi et al. 2014; Jian et al. 2018; Zhang et al. 2022). Several scientists have investigated triterpenoids in loquat and its byproducts. Jiang et al. (2016) determined that the content of triterpenoic acid varies in different species of loquat flowers. The content of three triterpenoids in loquat flowers was determined by HPLC, and it was found that the total content of triterpenoids in loquat flowers was 0.439%, of which corosolic acid, ursolic acid, and oleanolic acid accounted for 19.1%, 65.6%, and 15.3%, respectively. Most of the triterpenoids in the pericarp and pulp of loquat belong to ursolic acid derivatives and oleanolic acid derivatives (Xue et al. 2022). Jian et al. (2018) found that some differences existed in the triterpenoid content in different types of loquat leaves. The triterpenoid content of the fallen leaves was significantly higher than that of the mature leaves, which might result from the continuous accumulation of triterpenoids with the continuous development of loquat leaves. The triterpenoids in loquat are highly valued for their abundant biological activities and their wide range of health benefits. The research on these terpenoids not only provides a scientific basis for the development and utilization of loquat and its byproducts but also opens up new opportunities for the pharmaceutical and food industries. With further research and technological advances, the triterpenoids will play an important role in future health foods. In addition, ongoing research will continue to reveal their potential mechanisms and efficacy in treating various diseases, which is expected to contribute more to human health Figure 2.

### 2.3 Volatile Compounds

Loquat fruits, flowers, and leaves also contain a wide variety of volatile compounds, which contribute significantly to the unique aroma and potential health benefits. Studies have shown that the volatile compounds of loquat are mainly sesquiterpenes, monoterpenes, and aliphatic and aromatic compounds. Their chemical structures cover types such as alkanes, alkenes, aldehydes, ketones, alcohols, acids, and esters (Li et al. 2009).

Shen et al. (2023) found that the types and contents of volatile compounds in loquat flowers changed at different flowering stages. Zhang et al. (2008) identified 49 aroma compounds in loquat flowers by SPME‐GC–MS analysis, which accounted for 93.34% of the total volatile compounds. On the other hand, Lu et al. (2013) identified 84, 90, 69, and 73 volatile compounds in four different stages of loquat flowers. And Farina et al. (2020) identified 35 volatile compounds during the study of microwave effects on 3 different loquats. In Huang's study (2023), he identified 43 (20 aldehydes, 7 esters, 6 ketones, 1 alcohol, and 1 furan) volatile aroma compounds in loquat fruits by GC‐IMS. He also found that the content of ethyl methyl 3‐methyl butyrate, ethyl acetate, and dimethyl acetate were increased, and ethyl acetate and dimethyl ketone gradually decreased with storage time. The content of (E)‐2‐heptanol, heptanal, (E)‐2‐pentenal, 1‐penten‐3‐one, 3‐pentanone, and 2‐pentylfuran increased. These volatile compounds give loquat a unique aroma (Ayseli and İpek Ayseli 2016). Scientists have conducted studies on volatile compounds to gain insight into their potential effects. These studies help to reveal the active components of volatile compounds in loquat and provide important references for the development of loquat products.

### 2.4 Glycosides

Amygdalin is the main saponin component in loquat, which is distributed in various parts of loquat. It can be decomposed into hydrocyanic acid, phenylacetic acid, and glucose in the body. Among these, prussic acid is a toxic substance capable of inhibiting cell respiration and causing cell death. In recent years, amygdalin has been regarded as a promising natural substance with diverse anticancer effects (Liczbiński and Bukowska 2018). In addition, amygdalin can be used to treat respiratory diseases such as cough and asthma (Todorova et al. 2017). However, there are still some urgent and serious problems related to toxicity due to its cyano fraction as well as poor oral bioavailability (Spanoudaki et al. 2023). The results determined by Zhou et al. (2007) through HPLC indicated that the content of amygdalin in loquat flowers was in the range of 1.23–1.56 mg/g. There are relatively few studies on amygdalin in loquat and its byproducts, and it is anticipated to continue to be studied by scientists. Amygdalin has numerous potential applications. Further studies and clinical trials will assist in revealing the application potential of amygdalin in various fields and offer a more scientific basis for its future development and utilization.

## 3 Extraction and Separation of Biologically Active Substances

**FIGURE 3:**
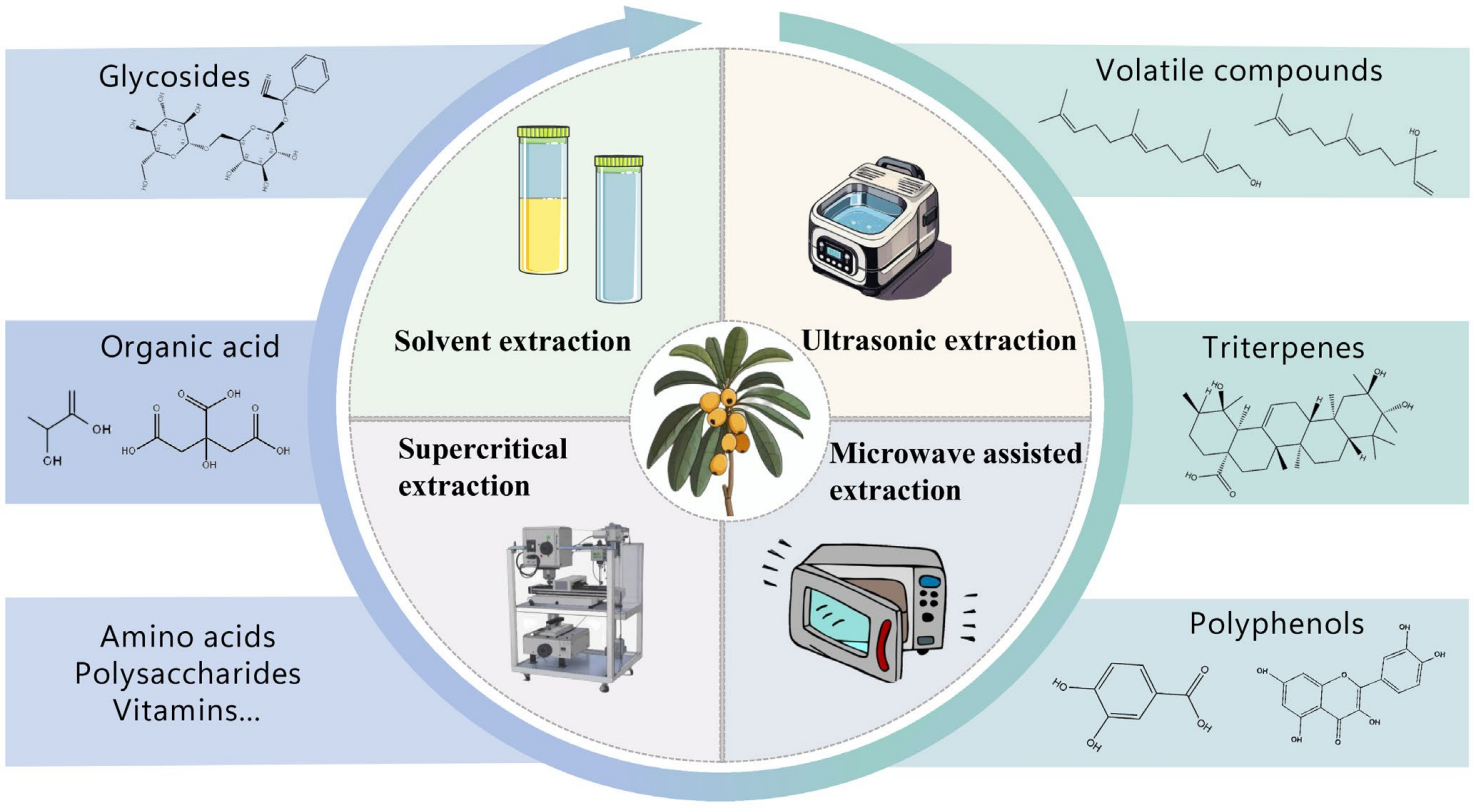
Extraction of active substances from loquat and its byproducts.

Loquat has numerous bioactive substances, and scientists have conducted in‐depth studies on its extraction methods (Figure 3). Traditional extraction methods mainly include water extraction and organic solvent extraction, which have been widely used in long‐term practice (Sharma et al. 2014; Seong et al. 2018; Wang et al. 2022). With the continuous development of science and technology, new technologies such as ultrasonic‐assisted extraction, supercritical fluid extraction, and microwave‐assisted extraction are also gradually attracting attention (Table 3). These modern methods have advantages in terms of extraction efficiency, speed, and purity of components, providing more choices and opportunities for loquat bioactive extraction research.

**TABLE 3:** Different methods, advantages and limitations of active substance extraction from loquat.

Method of extraction	Advantage	Limitation	Considerations	References
Soxhlet, maceration and distillation	The solvent is economical and easy to obtain; Simple to operate and easy to control the extraction process; Suitable for the extraction of various raw materials.; High extraction efficiency	Extraction takes long time and yields are low; High solvent usage raises costs; Heat‐sensitive compounds may decompose during extraction; Some processes are complicated; Extract properties vary; Residual solvents remain in the extract	It is suitable for preliminary extraction of plant components, large‐scale extraction, and is used for more stable substances.	Delfanian et al. (2015a, 2015b), Delfanian et al. (2016); Mlyuka et al. (2016)
Deep eutectic solvents	Designability; Environmentally friendly solvents; Easy to manufacture, lower cost; Good thermal stability, low volatility and low vapor pressure	Water stability needs to be studied. Easy to emulsify under some conditions; It may present solid state and high viscosity at room temperature	Combination and optimization of specific des, study of cytotoxicity, and specific extraction conditions	Bajkacz and Adamek (2017); Cao et al. (2020); (Huang, Guo, et al. 2023); (Huang, Liu, et al. 2023); Aktaş and Kurek (2024)
Ultrasonic extraction	High extraction efficiency; The extraction time was short. Low extraction temperature; Wide adaptability; Be used in combination with other methods	The restriction of ultrasonic attenuation factors; Difficult for industrial application; Require specific procedures	Control the thermal effect; Adjusting ultrasonic frequency and energy; Optimization parameter	Han et al. (2020); Xie et al. (2019); Gómez‐Cruz et al. (2021); Xue et al. (2022); Cisneros‐Yupanqui et al. (2023); Pawłowska et al. (2023)
Microwave assisted extraction	Save solvent; The process is simple; Wide application range; Safety and environmental protection	Not suitable for some substances with poor thermal stability; Reduce selectivity; The material is required to have good water absorption	Select a solvent that is transparent or translucent to microwave, and has a certain polarity; The selection of microwave radiation conditions; Consideration of moisture or humidity in the material	Han et al. (2020); Lei et al. (2019)
Supercritical extraction	High efficiency and yield; Fast extraction time and short production cycle; High security; Low temperature operation	High equipment cost; Cleaning is difficult; Limited ability to dissolve polar substances	The use of entrainment agent increases the difficulty of separating and recovering entrainment agent. Different extraction processes are required; According to the size of its ability to dissolve different substances to confirm whether it is suitable for use	Kawahito et al. (2008); Saravana et al. (2017); Tyśkiewicz et al. (2018); Escobedo‐Flores et al. (2018); Rodrigues et al. (2023)

### 3.1 Pre‐Treatment of Samples

The drying methods have varying and significant effects on bioactive compounds (Fu et al. 2020). Therefore, the selection of appropriate drying methods plays a crucial role in maintaining the stability of actives in food. Ullah et al. (2018) compared the differences between loquat fruits under traditional sun drying and mechanical hot air drying, and the study revealed that hot air drying with flat plate solar collectors is more suitable for the drying process and can obtain good texture and dried loquats with more antioxidants. Further, Li et al. (2019) investigated the effect of vacuum drying on loquat fruit polyphenols to demonstrate the feasibility of vacuum drying in loquat and by‐product processing. The results showed that vacuum drying at 140°C was superior to vacuum drying at 70°C, where the polyphenol content and antioxidant activity were high. A prolonged drying time might result in the oxidation or volatilization of flavor substances. This, in turn, can accelerate the evaporation of water at high temperature and enhance the drying efficiency. Meanwhile, the vacuum environment reduces the impact of oxygen, thus decreasing the oxidation reaction. Nevertheless, high temperature could potentially damage certain heat‐sensitive nutrients. Although the application of vacuum drying technology in processing loquat and its by‐products has the merits of short processing time and easy operation and can provide technical and theoretical support for the production of high‐quality and high‐nutritional value dried loquat fruits, it still demands more meticulous selection and reliable experimental verification.

Microwave drying treatment is a relatively new dehumidification technology that can significantly reduce the time required to reach the set temperature, thus shortening the overall drying time. Farina et al. (2020) showed that microwave drying maintained better sensory properties when treating different loquat species. Although the loss of carotenoids in loquat was greater after drying, aldehydes showed less change in the dried product. To maintain the quality and nutritional value of loquat products, microwave drying is a viable option in loquat processing.

Freeze‐drying, as an efficient pretreatment method, can effectively retain bioactive compounds in the material. In López‐Lluch et al. (2020), freeze‐drying was superior to other methods in maintaining color stability and carotenoid content. Therefore, freeze‐drying can be regarded as a superior drying method that helps to preserve the quality and bioactive components of loquat.

The drying methods have markedly different effects on the bioactive compounds in loquat and its byproducts. The selection of appropriate drying techniques can not only maintain the stability of bioactive compounds in food products but also improve the sensory quality and nutritional value of the products. Therefore, the selection of the most suitable drying technique to maximize the retention of the bioactive components in loquat is essential to improve the quality and health benefits of processed loquat products. At present, numerous studies have demonstrated that freeze‐rying can effectively retain bioactive components, better preserve functional components, and offer better sensory quality while reducing the growth of microorganisms (Lachowicz et al. 2019; Lan et al. 2020; Bhatta et al. 2020; Wang, Wen, et al. 2023; Wang, Zheng, et al. 2023; Li et al. 2024). Despite its high energy consumption, vacuum freeze‐drying is a recommended approach from the perspective of nutrition and quality preservation.

### 3.2 Isolation and Purification of Bioactive Compounds

#### 3.2.1 Solvent Extraction

In recent years, scientists have conducted in‐depth studies on solvent extraction, which provides important theoretical and practical support for efficiently extracting and utilizing bioactive substances. Organic solvent extraction is a commonly used chemical separation method that can effectively extract target compounds from complex mixtures. Delfanian et al. (2015a, 2015b) showed that the ethanol‐water solvent had the highest extraction rate of phenolic and tocopherol compounds among the three solvents (ethanol, water, and ethanol‐water) for loquat pericarp and pulp extraction. Subcritical water extraction takes advantage of the fact that many of the unique properties of water at elevated temperatures can enhance the solubility of certain compounds and make them more easily separable from solid matrices. Then, Mlyuka et al. (2016) investigated the extraction of one of these triterpenoids using subcritical water extraction, and the results were generally consistent with model predictions. Subsequent studies by others (Delfanian et al. 2016) found that among the three techniques, ultrasound‐assisted, supercritical carbon dioxide, and solvent extraction, solvent extraction was the most effective method for extracting phenolic compounds from loquat pericarp extracts. These studies indicate that the traditional extraction method still holds a unique position in scientific research. However, its existing problems cannot be overlooked. Traditional water extraction and organic solvent extraction methods commonly encounter issues such as low extraction efficiency and difficulty in precisely controlling extraction conditions. This is especially true when extracting some active ingredients that are hard to dissolve, as these processes often demand long‐term heating or reflux, thereby leading to high energy consumption.

Simultaneously, the extract may contain a greater amount of impurities like proteins, polysaccharides, and tannins, which elevate the difficulty of subsequent purification steps. From an environmental perspective, a large quantity of organic solvents may be consumed during the extraction process, resulting in contamination.

Deep eutectic solvents (DES) are analogs of ionic liquids with the advantages of designability, low vapor pressure, recyclability, and biodegradability (Bajkacz and Adamek 2017). Conventional extraction methods are usually costly and involve toxic solvents, DES offers a promising and sustainable alternative for the extraction of active ingredients such as polyphenols from edible plants (Aktaş and Kurek 2024; Huang, Guo, et al. 2023). (Huang, Liu, et al. 2023) screened the most suitable hydrophobic solvents for the extraction of triterpenoids from loquat leaves from 57 designed DES using the COSMO‐SAC model. Under the optimized extraction conditions, the extraction rate of ursolic acid, the main component, reached 17.769 ± 0.799 mg/g. This was significantly higher than that of 80% ethanol extraction. Meanwhile, Cao et al. (2020) screened and evaluated 10 DES from 60 prepared natural deep eutectic solvents (NDES) with low viscosity and sufficient safety. The experimental results also showed that NDES not only improved the stability of phytochemicals but also enhanced biological activity. These findings provide an important experimental basis for the efficient extraction of active substances from loquat and its byproducts and also broaden the mindset of scholars for in‐depth research on the extraction methods of loquat bioactive compounds.

However, some deep eutectic solvents (DES) have several issues. First of all, the preparation costs can be high. Second, when an organic amine is used as a DES extractant, emulsification easily occurs, which will decrease the extraction efficiency and make the subsequent separation more difficult. Besides, some DES are unstable at high temperatures or under specific conditions.

Solvent extraction, a well‐established and reliable method, holds considerable significance in bioactive research. But the separation of bioactive components, metal ions, and organic compounds in DES is still in the exploratory stage, and research on its theoretical basis and separation mechanism is relatively insufficient. As technology advances and is further refined, it is anticipated that solvent extraction will increasingly offer opportunities for the effective extraction and application of bioactive compounds.

#### 3.2.2 Ultrasonic Extraction

Ultrasonic extraction is favored by researchers because of its high efficiency, low cost, and low energy consumption (Xie et al. 2019), and is widely used in food science and biomedical fields. Cisneros‐Yupanqui et al. (2023) showed that ultrasound‐assisted extraction can significantly increase the total phenolic content and antioxidant activity of the active compounds, which is a significant advantage over the traditional extraction methods. Therefore, ultrasonic extraction is considered a reliable and efficient extraction method with a wide potential for application.

Pawłowska et al. (2023) identified 35 different compounds from loquat fruits and leaves using ultrasonic ethanol extraction. This result fully exhibits the potential of ultrasound technology as a highly efficient extraction method. It is capable of extracting more active compounds in less time with less solvent. In addition, Xue et al. (2022) thoroughly investigated the effect of ultrasound‐assisted extraction on the total triterpenoid constituents in the pulp and peel of loquat and summarized the optimal extraction conditions. The total triterpenoid content reached 11.69 ± 0.25 and 13.92 ± 0.20 mg/g, respectively, under these optimized conditions. This result indicates that the extraction rate and purity of the target compounds can be significantly improved by precisely controlling the extraction parameters. This is important for commercial production and application. Furthermore, determining optimal extraction conditions helps standardize the production process and ensure consistent product quality and efficacy. This is not only valuable for scientific research but also provides a reliable reference for industrial applications.

To improve the extraction efficiency of phytoactives, ultrasound extraction is often used in combination with other extraction methods. Han et al. (2020) used ultrasound‐microwave assisted extraction to extract total triterpenoid acid, which was optimized by response surface methodology. The extract was then purified with X‐5 macroporous resin. The yield of total triterpenoic acid increased to 281.24 mg/g compared to 35.71 mg/g in unassisted extraction, which gave better results. This method combines the effects of ultrasound and microwave radiation and utilizes their physical and chemical effects to more efficiently facilitate the interaction of the solvent with the targeted components in the sample, thereby accelerating the extraction process. However, the advantages and disadvantages of this method have not been fully evaluated. Aspects such as experimental reproducibility, stability, and comparative analysis with conventional methods need to be considered. Gómez‐Cruz et al. (2021) used optimized ultrasound‐assisted water extraction (UAWE) and showed that using ultrasound improved the efficiency of extracting triterpene acids without grinding. Ultrasound‐assisted water extraction not only improves the extraction efficiency but also saves time and cost. This provides new ideas and methods for the study of the extraction of bioactive compounds from loquat and byproducts. By utilizing the action of enzymes, the cell wall can be disrupted more efficiently to release the active substances in the plant, thus improving the extraction efficiency. Durmus and Kilic‐Akyilmaz (2023) compared the bioactivities of conventional heat, enzyme, ultrasound, and ultrasound enzyme‐assisted extractions of phenolics, and the results showed that the content of phenolics in the enzyme‐assisted extractions increased two to four times compared to the heat‐assisted extractions. This suggests that by improving the extraction efficiency and increasing the yield of target compounds, enzyme‐assisted extraction methods offer significant advantages in the extraction of phenolics. Although numerous studies have demonstrated the potential benefits of ultrasonic extraction, it must be noted that the efficiency of this extraction method is influenced by multiple factors. Moreover, the high temperature generated during ultrasonic extraction might cause structural alterations and a reduction in the activity of some heat‐sensitive phytochemicals.

Taken together, ultrasonic extraction of bioactive compounds from loquat is a feasible approach. The study of these methods for extraction of these compounds from loquat and its byproducts not only helps to gain a deeper understanding of the extraction properties of loquat but also can play an important role in the field of plant extraction and pharmaceutical research.

#### 3.2.3 Supercritical Extraction

Supercritical Fluid Extraction (SFE) is a highly efficient separating technique that uses supercritical fluids as solvents to extract active compounds. This technique has been widely applied and developed in many fields due to its unique advantages. The bioactive substances in loquat are easily destroyed during purification and separation. As a mild extraction method, SFE has a wide research prospect. Kawahito et al. (2008) used CO_2_ as an extractant to extract amygdalin and β‐sitosterol from loquat seeds and found that changes in temperature and pressure would affect the yield of the products. The optimum conditions for amygdalin and the recovery of amygdalin were obtained at 80°C and 20 MPa. In addition, the non‐polar nature of CO_2_ can be affected by the addition of polar modifiers. Methanol, ethanol, water, and isopropanol are currently the most commonly used modifiers (Tyśkiewicz et al. 2018). The viability of ethanol (80% v/v) as a co‐solvent for the extraction of polyphenols during supercritical extraction with carbon dioxide has been substantiated in a related study (Escobedo‐Flores et al. 2018). As researchers progressed, they began to explore different types of modifiers. Saravana et al. (2017) found that sunflower seed oil, soybean oil, rapeseed oil, ethanol, and water, when used as co‐solvents for supercritical carbon dioxide extraction, improved the stability of fatty acids, antioxidants, and oils extracted from algae. The study of Rodrigues et al. (2023) showed that the yields and concentrations of individual triterpenes by sunflower seed oil exceeded those of Soxhlet extraction, with temperature being one of the most influential parameters on yield. All these studies provide a useful reference for the extraction of active compounds from loquat and byproducts. Although SFE holds great potential in extracting active substances, there are several issues during the process. For instance, the high‐pressure material system is not ideal, there is a lack of physicochemical data for various substances, and there is insufficient research on the nature of the interaction between the solute and the supercritical medium, which makes it impossible to establish a satisfactory correlation or prediction model. Simultaneously, the initial investment and maintenance costs of the equipment are too high, and the molecular structure, polarity, and other properties of the solutes limit the further expansion of SFE applications.

In conclusion, solvent extraction, ultrasonic extraction, and supercritical extraction have demonstrated their respective advantages in the extraction of bioactive compounds from loquat and its byproducts. High‐efficiency extraction can be achieved by optimizing the solvent extraction method. The introduction of deep eutectic solvent provides a new promising option for green and sustainable extraction. The extraction efficiency can be significantly improved by using ultrasound in combination with microwave and enzymatic techniques. On the other hand, supercritical extraction as a gentle extraction method can effectively prevent the destruction of the active compounds while extracting. These studies not only provide an effective method and an important reference value for the extraction of active compounds in loquat but also promote the development of its extraction technology.

## 4 Bioactivity and Mechanism of Loquat

Loquat of bioactive compounds have wide and good biological function, one of the most significant is its antioxidant, anti‐inflammatory and hypoglycemic effect. These functions are mainly attributed to the abundance of phenols and triterpenoids in loquat. These bioactive compounds act through a variety of targets and pathways of action. And they have been shown to have potential antioxidant, anti‐inflammatory and anti‐cancer properties. However, further studies on the specific molecular mechanisms of these effects are needed to better understand their application in disease prevention and treatment. Therefore, we summarized most of the active compounds in loquat and their mechanisms of action (Table 4), hoping to provide important reference values for research in the field of loquat and its by‐products.

**TABLE 4:** Activity and mode of action of components in loquat.

Active ingredient	Activity	Mode of action	Plant part	References
Phenolics	Antioxidant activity; Anti‐inflammatory activity; Antibacterial activity; Cardiovascular protection; Anti‐tumor activity	Inhibition of oxidase activity; Enhancement of antioxidant enzyme activity; Inhibition of inflammatory factors; Modulation of immune cells; Inhibition of glycosylated serum proteins; Inhibition of bacterial growth	Fruit, leaf, flower	Hanasaki et al. (1994); Li et al. (2007); Lü et al. (2009); Leopoldini et al. (2011); Mokdad‐Bzeouich et al. (2015); Chen et al. (2017); Lee et al. (2017); Beken et al. (2020); Ulusoy and Sanlier (2020); Lee and Jeong (2021); Sousa et al. (2021); Zhou and Huang (2023); Yan et al. (2023); Yan et al. (2024); Wang et al. (2024)
Terpenoid	The regulating effect on physiological function; Antibacterial and antiviral activity; Antioxidant activity; Anti‐inflammatory activity; Antitumor activity	Inhibition of inflammatory factors; Regulation of related enzyme activities; Inhibition of NO increase; Repair of insulin receptors; Regulation of TRPV1 and SIRT6/Nrf2 signaling pathways; Inhibition of cancer cells	Fruit, leaf, flower	Lin and Tang (2008); Lü et al. (2009); Huang et al. (2009); Yeh et al. (2010); Cha et al. (2011); Shin et al. (2017); Fu et al. (2019); Guo et al. (2020); Chen et al. (2021); Wu et al. (2021); Hyun et al. (2022); Khouya et al. (2022)
Vitamin C	Anti‐oxidation effect; Promote collagen synthesis; Enhance immunity; Involved in iron absorption and metabolism	Clearing free radicals; promoting iron absorption; Enhancement of antioxidant activity	Fruit, leaf, flower	Sanford et al. (2017)
Ursolic acid	Anti‐inflammatory effect; Anti‐oxidation effect; Antitumor effect; Immunoregulatory effect; Protective effect on skin	Reduces AKT/mTOR pathway signaling, inhibits MEK/ERK signaling pathway and IKK/NF‐κB pathway, regulates proliferation and apoptosis.	Fruit, leaf, flower	Yeh et al. (2010); Guo et al. (2020).
Vitamin E	Anti‐oxidation effect; Protective effect on cardiovascular system; A boost to the immune system	Protecting cell membranes; providing electrons	Fruit, leaf, flower	Wang (1999); Saito (2021)
Carotenoids	Anti‐oxidation effect; The role of the visual system; Immune system effects; Protective effect of cardiovascular system; Effects on skin health	Bursting of reactive oxygen species	Fruit, flower	Edge and Truscott (2018); Dhiman et al. (2022)
Nerolidol	Antibacterial activity; Anti‐inflammatory effect; Antioxidant activity; Effects on the nervous system	Reducing MCP‐1 secretion	Flower	Bastaki et al. (2021)
Linalool	Antibacterial activity; Anti‐inflammatory effect; Antioxidant activity	Inhibition of inflammatory factors; cell activation	flower	Kim et al. (2019)
Amygdalin	Antitumor activity; Immunoregulatory activity; Antioxidant activity; Anti‐inflammatory activity	Inhibition of inflammatory factors; reduction of integrin expression Inhibits PI3K enzymes; affects neurotransmitter release	Nucleus	Onogawa et al. (2009); Todorova et al. (2017); Liczbiński and Bukowska (2018); Shahat et al. (2023); Spanoudaki et al. (2023)

Abbreviations: AKT, Protein kinase B; ERK, Extracellular regulated protein kinases; IKK, IκB kinase; MCP‐1, Monocyte Chemoattractant Protein‐1; MEK, Mitogen‐activated protein kinase; mTOR, Mammalian target of rapamycin; Nrf2, Nuclear factor erythroid 2‐related factor 2; PI3K, Phosphatidylinositol 3‐kinase; SIRT6, Sirtuin 6; TRPV1, Transient Receptor Potential Vanilloid 1.

### 4.1 Antioxidant Activity and Mechanism of Action

**FIGURE 4:**
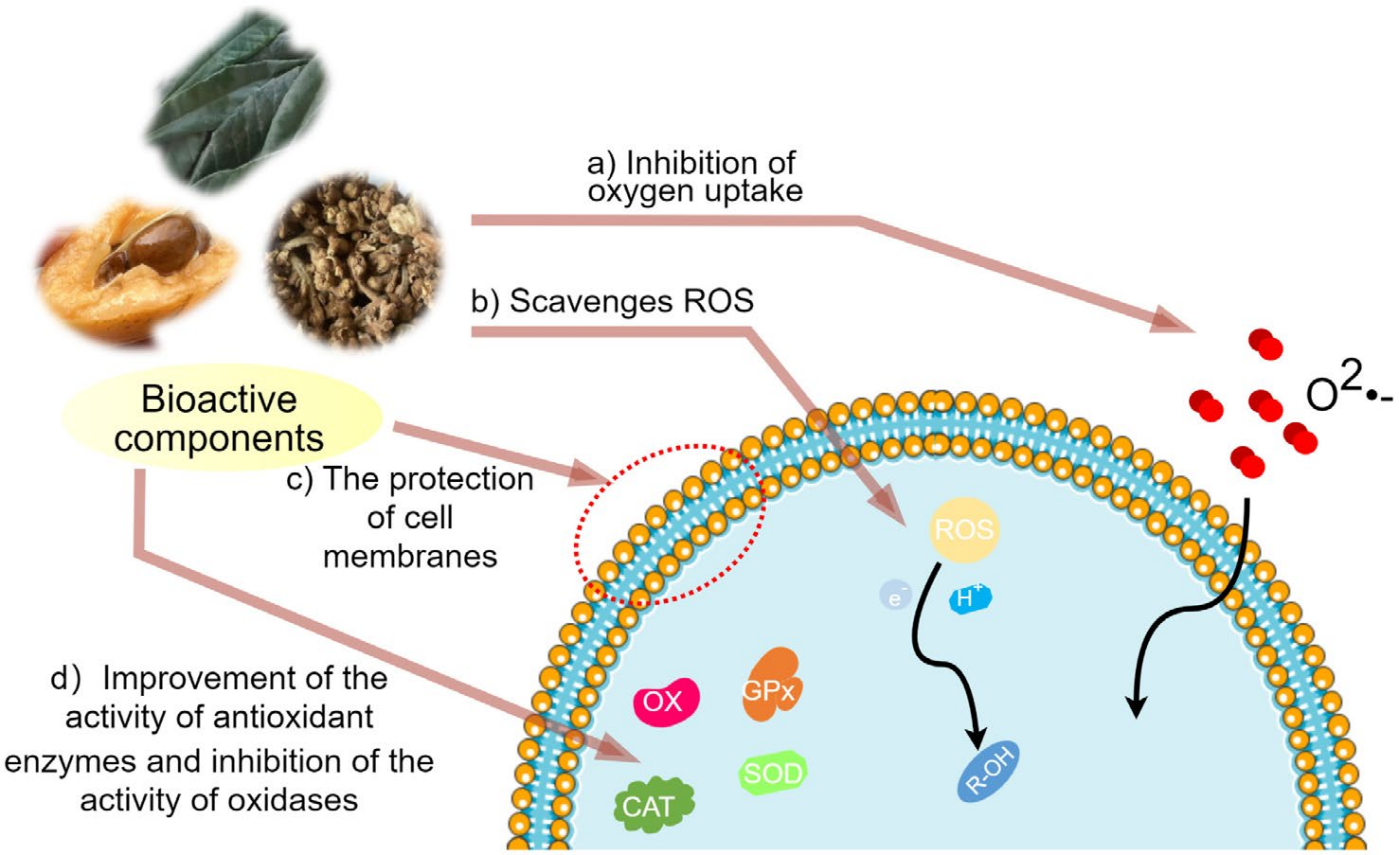
Antioxidant mechanism of active substances in loquat and its byproducts.

The negative effects of oxidative reactions on cells constitute an extensively researched area, encompassing peroxidation of cell membrane lipids, disruption of cellular integrity and fluidity, and the consequent inflammatory response. These effects accelerate cellular senescence and death and affect overall health. The extracts of loquats and its byproducts have garnered attention for their significant antioxidant activity (Figure 4). Studies have shown that they are effective against oxidative stress and free radical‐induced damage (Jung et al. 1999; Koba et al. 2007; Kim et al. 2011; Mokhtari et al. 2023).

The main sources of antioxidant activity in loquat and its byproducts are vitamin C, vitamin E, polyphenolic compounds, and carotenoids. These components safeguard cells from oxidative damage by scavenging free radicals within the body and inhibiting oxidative stress (Mokdad‐Bzeouich et al. 2015). Flavonoids, as a type of polyphenols, directly scavenge free radicals derived from superoxide or highly reactive oxygen species (Li et al. 2007). In addition, flavonoids can reduce the production of free radicals by inhibiting the activity of xanthine oxidase, thereby protecting cells (Hanasaki et al. 1994; Zhou and Huang 2023). Components in loquat and its byproducts also reduce the production of lipid peroxidation products such as malondialdehyde, further protecting cell membranes (Ulusoy and Sanlier 2020). Polyphenols enhance the endogenous antioxidant system and maintain intracellular redox balance by promoting the activity of antioxidant enzymes such as catalase, glutathione peroxidase, and superoxide dismutase (SOD) (Sousa et al. 2021; Yan et al. 2023). In the meantime, polyphenols can chelate metal ions, inhibit the production of free radicals, prevent oxidative stress, or reduce peroxidation, and protect cells from damage (Ulusoy and Sanlier 2020; Leopoldini et al. 2011).

Vitamin C is abundant in loquat, which not only neutralizes free radicals in the body and reduces damage to cell membranes, proteins, and DNA, but also promotes iron absorption and aids in oxygen transport (Sanford et al. 2017). On the other hand, Vitamin E forms a protective layer on the cell membrane to prevent free radical damage and stabilizes free radicals by donating electrons to reduce their activity. This protects cells from oxidative damage and maintains the integrity and function of the cell membrane (Wang 1999; Saito 2021). Carotenoids, such as β‐carotene and β‐cryptoxanthin, make up more than half of the colored carotenoids in loquats (Dhiman et al. 2022). As precursors of vitamin A, carotenoids can inhibit highly reactive single linear oxygen species and protect cells from oxidative damage (Edge and Truscott 2018). In summary, the antioxidant properties of the extracts from loquats and its byproducts provide a multifaceted mechanism to protect cells from oxidative damage. This is important for maintaining health and preventing diseases associated with oxidative stress.

### 4.2 Anti‐Inflammatory Activity and Mechanism of Action

**FIGURE 5:**
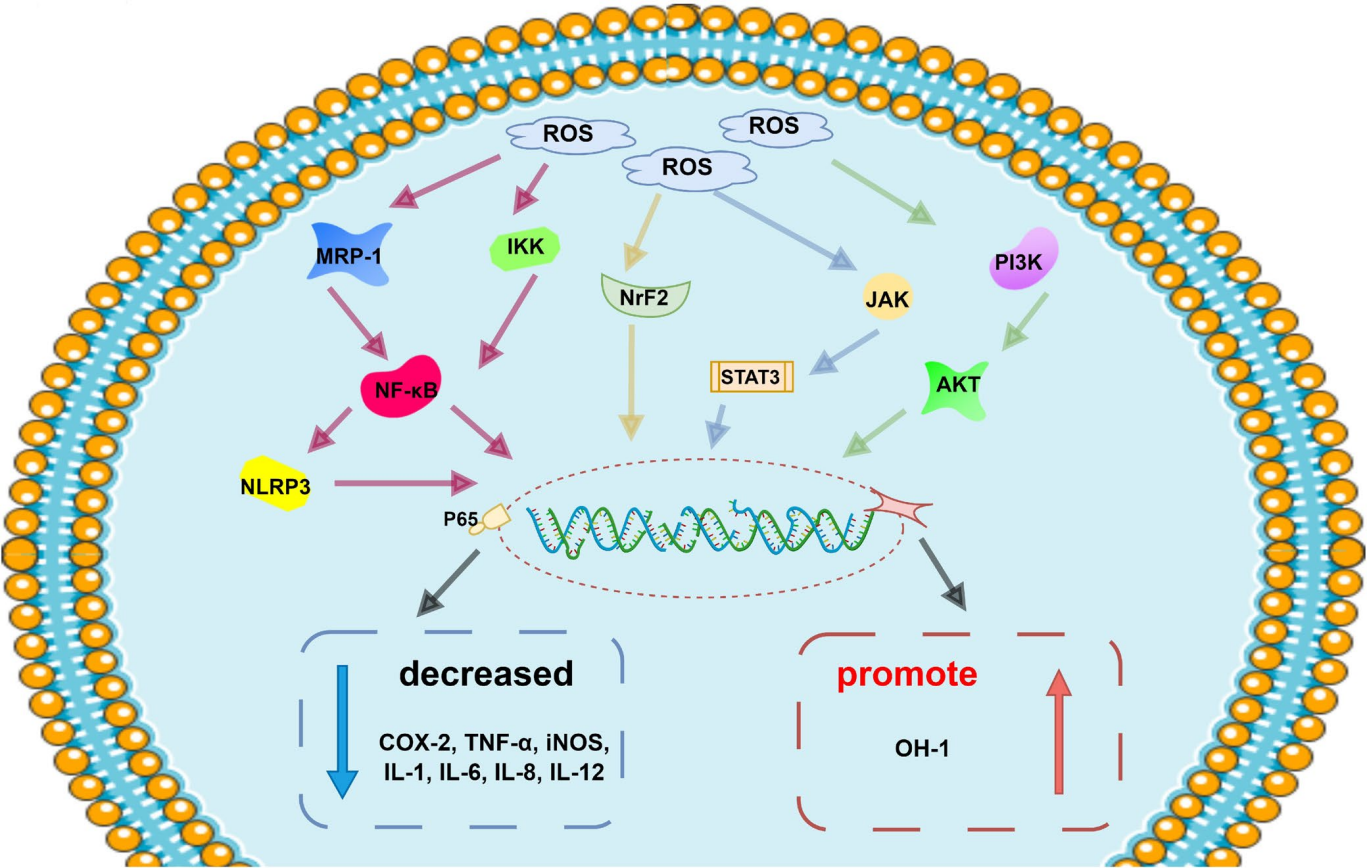
Anti‐inflammatory mechanism of active substances in loquat and its byproducts.

Inflammatory response is a natural response of the human body to injury or infection, which helps to eliminate pathogens and repair damaged tissues. However, when the inflammatory response is excessive or prolonged, it can lead to tissue damage and dysfunction. Long‐term chronic inflammatory responses have also been implicated in the development and progression of many diseases. These include cardiovascular disease, diabetes, and arthritis. The anti‐inflammatory effects of the bioactive compounds in loquat are currently being extensively studied (Figure 5). The anti‐inflammatory mechanism of bioactive compounds involves multiple pathways. These include regulation of pro‐inflammatory factors, inhibition of regulatory enzymes, and inactivation of transcription factors (Zar et al. 2014; Kim et al. 2023).

The active substances in loquat and its byproducts can inhibit the secretion of interleukin‐6 (IL‐6) and tumor necrosis factor‐α (TNF‐α) and increase the secretion of IL‐10, which can inhibit the inflammatory response (Lin and Tang 2008). Cha et al. (2011) obtained similar results and found that the loquat leaves can downregulate the activation of nuclear factor‐κB (NF‐κB) to inhibit the production of NO and the expression of iNOS and COX‐2. In a subsequent investigation (Huang et al. 2009), Huang et al. first ascertained that triterpenoid acid in loquat inhibited the increase in NO, expression of inducible iNOS, and phosphorylation of p38 MAPK in macrophages (AMs), which led to alleviating and lessening inflammation. The flavonoids in loquat inhibited the expression of IL‐1β, IL‐6, IL‐8, and thymic stromal lymphopoietin, while increasing the expression of catalase, superoxide dismutase‐1, glutathione peroxidase, and IL‐10, which in turn alleviated the symptoms associated with atopic dermatitis and accelerated wound healing through NF‐κB and ERK1/2 MAPK (Beken et al. 2020). Lee et al. came to the same conclusion in the lipopolysaccharide (LPS)‐stimulated RAW264.7 macrophage model experiment (Lee et al. 2017). The active compounds in loquat and its byproducts not only intervene in signaling pathways to alleviate inflammation but also regulate T cell activation by inhibiting activated T cells and MRP‐1 activity in vivo to alleviate atopic dermatitis (Lee and Jeong 2021). Meanwhile, flavonoids such as rutin and kaempferol can also inhibit immunoglobulin E (IgE) production, which leads to inhibiting the activation of mast cells (Yan et al. 2024). They effectively regulate the immune response in animals with atopic dermatitis.

Linalool can also significantly reduce the number of eosinophils, Th2 cytokines, and IgE levels induced by ovalbumin (OVA) exposure. It can also effectively downregulate the activities of mitogen‐activated protein kinases (MAPKs) and NF‐κB; In the meantime, nerolidol treatment can reduce the secretion of monocyte chemotactic protein‐1 (MCP‐1), nerolidol can effectively counteract airway inflammation and mucus hypersecretion (Kim et al. 2019), and it has also been shown that nerolidol treatment significantly reduces calreticulin and pro‐inflammatory cytokines to alleviate inflammatory bowel disease (Bastaki et al. 2021).

The research on the anti‐inflammatory effects of loquat's bioactive compounds is still in progress, and more in‐depth studies are requisite to comprehensively understand and exploit their potential. Undoubtedly, this will contribute to the development of novel anti‐inflammatory strategies and therapeutics, conferring benefits upon human health.

### 4.3 Hypoglycemic Activity and Mechanism of Action

**FIGURE 6:**
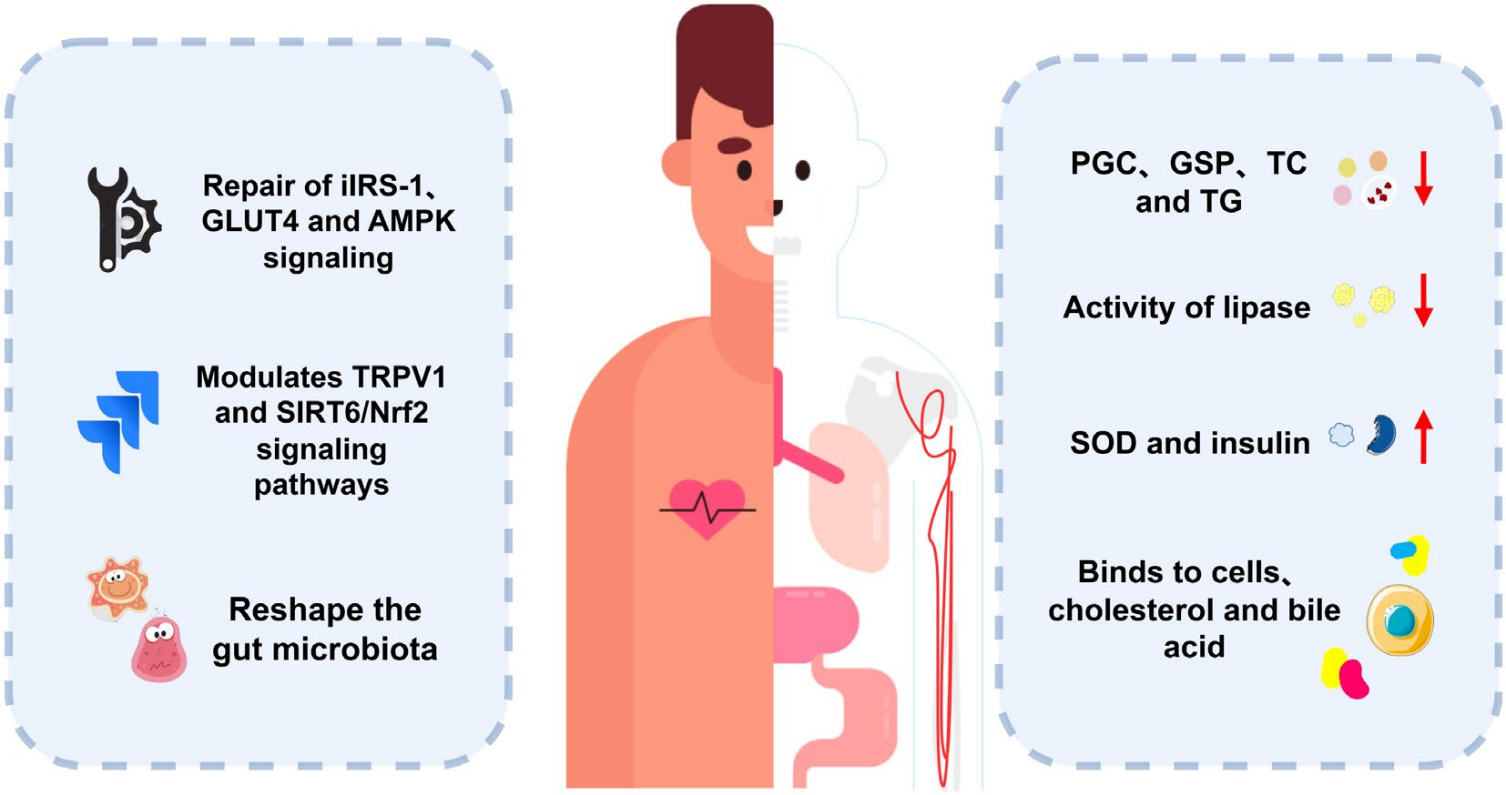
Hypoglycemic mechanism of active substances in loquats and its byproducts.

Diabetes is a global health concern. In recent years, several advancements have been achieved in diabetes research with technological progress and a deeper comprehension of the disease mechanism. Particularly, scientists have revealed the pharmacological mechanism of action of the active compounds in loquat through numerous experimental studies (Figure 6). This presents new prospects for the treatment of diabetes.

The α‐amylase and α‐glucoside are key enzymes involved in the enzymatic process of digesting starch and glycogen (Sales et al. 2012). Polyphenolic compounds in loquat can effectively inhibit the activities of α‐amylase and α‐glucosidase. Chen et al. (2017) demonstrated that the ethanolic extract of loquat leaves had a strong inhibitory effect on α‐glucosidase, which may be caused by the binding of the hydroxyl group of phenolic acid to tryptophan. Similarly, a better inhibitory effect on α‐amylase was obtained by Vyas (2019) using loquat extracts. Lü et al. (2009) found that the flavonoids in loquat leaves significantly reduced plasma glucose concentration, glycated serum protein, total cholesterol, and triglycerides, and significantly increased SOD activity and serum insulin level, indicating a hypoglycemic activity. Hyun et al. (2022) further investigated the mechanism loquat leaf extract in treating diabetes. They hypothesized that the LKB1/AMPK/FOXO3 signaling pathway is a potential target responsible for the beneficial effects of loquat leaf extract and its major component of ursolic acid. Loquat leaf extracts have potent cell‐binding, cholesterol‐binding, and bile acid‐binding properties (Fu et al. 2019). This may be due to their molecular weight, molecular weight distribution, and degree of deposition. There was also a dose‐dependent effect of aqueous loquat leaf extract on ameliorating hyperglycemia, and insulin resistance (Khouya et al. 2022). These studies provide important clues to elucidate the pharmacological mechanism of action of loquat compounds. They also offer potential hope for the treatment of diabetes mellitus.

Potential therapeutic effects of total sesquiterpene glycosides (TSGs) from loquat on insulin resistance (IR) induced by a high‐fat diet. Total sesquiterpene glycosides repaired insulin receptor substrate‐1 (IRS‐1)/glucose transporter 4 (GLUT4) and AMP‐activated protein kinase (AMPK) signaling. They improved glucose and lipid metabolism and prevented lipid accumulation in the liver. Meanwhile, TSG can improve IR in vivo by enhancing IRS‐1/GLUT4 signaling and AMPK activation, as well as by modulating transient receptor potential vanilloid 1 (TRPV1) and the signaling pathway of sirtuin‐6 (SIRT6)/nuclear factor erythroid 2‐related factor 2 (Nrf2) (Wu et al. 2021). Nonalcoholic fatty liver disease is strongly associated with type 2 diabetes mellitus. Triterpene glycosides from loquat leaves can remodel the mouse gut microbiota by improving insulin resistance, modulating insulin signaling, and inhibiting the cytochrome P450 2E1 (CYP2E1) and NOD‐like receptor family pyrin domain‐containing 3 (NLRP3), ameliorating type 2 diabetes (Chen et al. 2021).

The considerable potential of natural plant resources for food and drug development, which offers hope for the treatment of diabetes and related metabolic diseases. The active ingredients of loquat and its byproducts have shown potential anti‐diabetic effects. We expect more breakthroughs in this field, bringing better therapeutic options and improved quality of life for diabetics, as scientific research and the application of new technologies continue to advance.

### 4.4 Other Activities

In addition to its antioxidant, anti‐inflammatory, and hypoglycemic effects, loquat has a wide array of other functional activities. For example, the polyphenols in loquat can have a significant inhibitory effect on the growth and activity of bacteria and fungi (Shen et al. 2021; Zhang et al. 2022). Studies have indicated that polyphenol extracts affect their normal activity by enhancing the permeability of microbial cell membranes. This can lead to leakage of intracellular proteins, depolarization of cell membranes, decrease in intracellular ATP content, increase in cell membrane potential, and leakage of cell contents (Wang et al. 2024).

Meanwhile, loquat leaf extract has an inhibitory impact on the metabolic activation of mutagens and carcinogens, which reduces the amount of mutagen‐induced reductants and protects DNA from electrophilic metabolites (Mokdad‐Bzeouich et al. 2015). The compounds such as flavonoids, coumarins, and tannins in the aqueous and total flavonoid extracts of loquat leaves have anti‐genotoxic and anti‐mutagenic activities. These activities may be mediated by multiple mechanisms such as interfering with the metabolism of mutagens and protecting DNA.

Moreover, compounds in loquat leaves can restrain the generation of osteoclasts. They down‐regulate the expression of mature microRNA let‐7 g‐5p to down‐regulate XPO5 during osteoclast differentiation, and thus alleviate bone resorption diseases (Tan et al. 2019). Shin et al. (2017) found that the ethanolic extract of loquat pericarp inhibited the proliferation of EJ bladder cancer cells through the p27KIP1 factor. Progressive studies have shown that ursolic acid in loquat can reduce the phosphorylation of protein kinase B (AKT)/ mammalian target of rapamycin (mTOR) pathway signaling, inhibit Raf/mitogen‐activated protein kinase kinase (MEK)/Extracellular Regulated Protein Kinases (ERK) signaling pathway and IKK/NF‐κB pathway, regulate proliferation and apoptosis, and achieve antitumor effects (Yeh et al. 2010; Guo et al. 2020). Amygdalin in loquat was also able to increase the expression of Bax protein and caspase‐3 and decrease the expression of anti‐apoptotic BcL‐2 protein (Liczbiński and Bukowska 2018). Related studies have shown that loquat extracts have anti‐allergic activity and can prevent damage to the heart (Onogawa et al. 2009; Shahat et al. 2023).

Loquat fruit extract significantly reduces hepatic endotoxin levels and improves intestinal barrier function in mice by modulating several molecular pathways (Li et al. 2019). It maintains the normal colonic Firmicutes/Bacteroidetes ratio in mice through interactions with tight junction proteins (ZO‐1 and occludin), mucus, and colonic immune responses and the relative abundance of Veillonella. This study suggests that loquat fruit extract may have a potential role as a nutritional agent for liver protection.

The diverse and remarkable functional activities of loquat conspicuously illuminate its substantial potential in numerous domains of health and medicine. The continuous research on the mechanisms and applications of these activities is indispensable for fully unleashing the benefits that loquat can provide to enhance human health and treat diverse diseases. As our comprehension intensifies, loquat is likely to evolve into an increasingly precious resource in the quest for superior healthcare.

## 5 Application of Bioactive Compounds in Loquat

The bioactive compounds in loquat have a wide range of potential applications in the fields of food, pharmaceuticals, nutrition, and cosmetics. The extensive utilization of loquat kernels as a source of non‐conventional starch holds great potential for industrial and food applications. It has been widely investigated for its use as a major macromolecule in industrial thickeners, gelling agents, and biodegradable and edible films, positioning it as a promising alternative to conventional starch. In the study by Kong et al. (2023), t the physicochemical properties of starch from different loquat species were found to be significantly different, highlighting the value of selecting appropriate loquat species for industrial production and scientific research. The porous starch prepared by researchers, extracted from discarded loquat kernels as described by Li et al. (2023), demonstrated favorable loading rates and thermal stability. Additionally, the loquat starch‐based film prepared by Costa et al. (2022) exhibits excellent antioxidant and preservation capabilities, which provides new ideas for the development of food packaging preservation. Furthermore, studies on the synthesis of loquat starch‐based nano delivery systems (Singh et al. 2019) and silver nanoparticles (Yu et al. 2019) demonstrated their remarkable antioxidant, anti‐diabetic, and degradation stability effects, providing new opportunities for medical innovation. In order to fully utilize the waste from loquat processing, researchers prepared activated carbon by carbonizing loquat kernels with CO_2_ and conducted characterization studies (Plaza‐Recobert et al. 2017). While research on the structural and morphological characteristics of loquat kernel starch is still limited, this deficiency indicates that our understanding of loquat starch remains superficial, thereby constraining its industrial potential. Concurrently, the current state of processing technology is immature, which may result in reduced efficiency and cost‐effectiveness. Therefore, further research is essential to refine the processing techniques and to create value‐added products suitable for mass production. Moreover, the mechanism of modified starch in loquat requires additional investigation, particularly concerning the interactions between starch and other food components, as well as their collective impact on the structural properties of starch.

Current research on loquat extract encompasses a broad spectrum of areas, including pharmacological effects, extraction methodologies, varietal distinctions, biological activities, and regulation of biorhythms. These studies underscore the extract's potential applications in the fields of medicine, food science, and beyond. Fe_3_O_4_NPs and Cu particles synthesized using loquat extract showed excellent antibacterial activity (Joharian et al. 2022), which is an important reference for the development of nanomaterial‐based antimicrobial agents. The ethanolic extract of loquat leaves was found to be non‐irritating to the skin and eyes in animal replacement tests. It also significantly inhibited melanogenesis (Seong et al. 2019). Huang et al. (2022) isolated a galacturonic acid (GalA)‐containing polysaccharide from loquat leaves, which showed potential anti‐inflammatory and antioxidant effects. This provides a valuable reference for future drug development. In the study by Shahat et al. (2023), rats given 100 mg/kg and 200 mg/kg doses of loquat leaf extract showed a positive effect on carbon tetrachloride intoxication. The study of the antibacterial activity against various pathogens and its biological activity on human skin cells of loquat leaf extract provides an important basis for the development of natural preservatives and skincare products. They also make a valuable contribution to the future development of related fields. Reasonable development and utilization of bioactive substances in loquat can contribute to social and economic development and human health. However, several limitations are evident in the current research on loquat extracts. Firstly, significant variations exist in the water and alcohol extracts of loquat leaves from the same provenances, yet research into these differences is inadequate, impacting the quality control and application of loquat extracts. Secondly, deficiencies persist in the investigation of the pharmacological effects, the identification of active components, their mechanisms of action, and potential synergistic effects of loquat leaves. This necessitates further research to elucidate the chemical constituents and biological activities of loquat leaves. Thirdly, the majority of research remains confined to the laboratory stage, with a notable absence of large‐scale industrial application studies.

## 6 Conclusion and Prospect

Loquats are characterized by their extensive geographical distribution, high yield, and richness in diverse bioactive compounds. The fruit, leaves, and flowers of the loquat ( *Eriobotrya japonica* Lindl.) are recognized as novel food ingredients, embodying both medicinal and edible properties. They are classified as food‐medicine homologues, possessing significant therapeutic or adjunctive therapeutic potential. Consequently, loquat extracts and by‐products are poised to be utilized in food, pharmaceuticals, cosmetic products, and numerous other sectors, potentially serving as a reliable and high‐quality source of bioactive ingredients for functional foods. These related products and extracts could serve clinical roles beyond nutrition, preceding pharmaceuticals, while functioning as nutrients to prevent or mitigate the effects of certain diseases. Moreover, the reprocessing and repurposing of loquat and its by‐products exemplify an environmentally conscious approach to converting waste into valuable health products. By developing innovative and high‐value food ingredients, the potential impact of loquat by‐products on agriculture, nutrition, and the food industry could be substantial, ultimately benefiting human health.

To enhance the reliable and effective utilization of loquats and their by‐products, further investigation into their composition, safety, and synergistic effects is imperative. Firstly, to ensure their safe application in the food industry, it is essential to establish that they are safe, reliable, and devoid of adverse effects on human health. Therefore, a more comprehensive in vivo evaluation of the toxicity of loquat by‐products and their extracts, as well as their impact on organs such as the heart, lungs, liver, and kidneys, and on intestinal microbiota, is warranted. Secondly, while numerous studies have demonstrated the beneficial activities of loquats and their by‐products, the active constituents remain unidentified due to the presence of a multitude of compounds. Hence, more in‐depth laboratory studies and clinical trials are necessary to pinpoint the specific phytochemicals in loquats and by‐products that contribute to these biological activities and to elucidate their protective mechanisms. For instance, identifying constituents with antioxidant or anti‐inflammatory properties and understanding their potential mechanisms of action provides a robust theoretical foundation for the future development of health foods aimed at preventing or treating related diseases, playing a crucial exploratory role. Concurrently, the extraction and purification processes for loquats and their by‐products are not yet optimized. There is a need to refine extraction and separation techniques to enhance efficiency, purity, and stability. Thirdly, loquats and their by‐products contain a complex array of diverse components, and a characteristic of plant extracts is that crude extracts often exhibit higher efficacy than their isolated single components. This may be due to synergistic interactions among these ingredients, thereby enhancing overall biological activity and therapeutic efficacy. Therefore, a thorough understanding of the synergies between various plant constituents is essential for the development of health foods with augmented activity. Lastly, achieving sustainable utilization of loquat resources, exploring environmentally friendly and green production methods, and fostering the sustainable growth of the loquat industry are also vital research avenues. In conclusion, loquats are not only a delectable fruit but also a natural treasure with considerable value, warranting further exploration.

## Author Contributions

**Xiaofeng Liu:** conceptualization (equal), funding acquisition (equal), methodology (equal), project administration (equal), supervision (equal), writing – review and editing (equal). **Xiulei Cai:** data curation (equal), methodology (equal), writing – original draft (equal), writing – review and editing (equal). **Zhiyu Chen:** software (equal), writing – review and editing (equal). **Yao Zhang:** writing – review and editing (equal). **Hao Zhong:** conceptualization (equal), methodology (equal). **Rongfa Guan:** funding acquisition (equal), project administration (equal), supervision (equal), writing – review and editing (equal).

## Conflicts of Interest

The authors declare no conflicts of interest.

## Data Availability

No data was used for the research described in the article.
